# RIPK1/RIPK3 promotes vascular permeability to allow tumor cell extravasation independent of its necroptotic function

**DOI:** 10.1038/cddis.2017.20

**Published:** 2017-02-02

**Authors:** Kay Hänggi, Lazaros Vasilikos, Aida Freire Valls, Rosario Yerbes, Janin Knop, Lisanne M Spilgies, Kristy Rieck, Tvisha Misra, John Bertin, Peter J Gough, Thomas Schmidt, Carmen Ruiz de Almodòvar, W Wei-Lynn Wong

**Affiliations:** 1 Institute of Experimental Immunology, University of Zurich, Zurich, Switzerland; 2 Biochemistry Center, Heidelberg University, Heidelberg, Germany; 3 Department of General, Visceral and Transplantation Surgery, Heidelberg University, Heidelberg, Germany; 4 Pattern Recognition Receptor Discovery Performance Unit, Immuno-Inflammation Therapeutic Area, GlaxoSmithKline, Collegeville, PA, USA

## Abstract

Necroptosis is an inflammatory form of programmed cell death requiring receptor-interacting protein kinase 1, 3 (RIPK1, RIPK3) and mixed lineage kinase domain-like protein (MLKL). The kinase of RIPK3 phosphorylates MLKL causing MLKL to form a pore-like structure, allowing intracellular contents to release and cell death to occur. Alternatively, RIPK1 and RIPK3 have been shown to regulate cytokine production directly influencing inflammatory immune infiltrates. Recent data suggest that necroptosis may contribute to the malignant transformation of tumor cells *in vivo* and we asked whether necroptosis may have a role in the tumor microenvironment altering the ability of the tumor to grow or metastasize. To determine if necroptosis in the tumor microenvironment could promote inflammation alone or by initiating necroptosis and thereby influencing growth or metastasis of tumors, we utilized a syngeneic tumor model of metastasis. Loss of RIPK3 in the tumor microenvironment reduced the number of tumor nodules in the lung by 46%. Loss of the kinase activity in RIPK1, a member of the necrosome also reduced tumor nodules in the lung by 38%. However, the loss of kinase activity in RIPK3 or the loss of MLKL only marginally altered the ability of tumor cells to form in the lung. Using bone marrow chimeras, the decrease in tumor nodules in the *Ripk3*^*−/−*^ appeared to be due to the stromal compartment rather than the hematopoietic compartment. Transmigration assays showed decreased ability of tumor cells to transmigrate through the vascular endothelial layer, which correlated with decreased permeability in the *Ripk3*^*−/−*^ mice after tumor injection. In response to permeability factors, such as vascular endothelial growth factor, RIPK3 null endothelial cells showed decreased p38/HSP27 activation. Taken together, our results suggest an alternative function for RIPK1/RIPK3 in vascular permeability leading to decreased number of metastasis.

Cell death is considered to be one of the hallmarks of malignancy – either by upregulation of anti-apoptotic genes or downregulation or silencing of pro-apoptotic genes.^1^ Necroptosis is a programmed cell death pathway triggered by tumor necrosis factor (TNF), Fas, Toll-like receptor ligands and type I interferon upon loss or inhibition of caspase-8.^2, 3, 4, 5, 6^ Understanding whether necroptosis is part of the cell death hallmark of malignancy has become an area of intense research.

Receptor-interacting protein kinase 1 (RIPK1), a multifunctional protein that contains an N-terminal Ser/Thr kinase domain, is known to be a critical regulator at the decision point of cytokine induced NF-kB activation for survival or cell death by either apoptosis or necroptosis.^7^ Necroptosis is triggered by RIPK1 interaction with RIPK3, RIPK3 dimerization, autophosphorylation of RIPK3 and subsequent phosphorylation of mixed lineage kinase domain-like protein (MLKL).^8, 9, 10^ Upon phosphorylation, MLKL is believed to form a pore complex that can compromise cell membrane integrity^11, 12^ resulting in the release intracellular contents. The result is an inflammatory response to the release of danger-associated molecular patterns and/or the direct regulation of cytokines by RIPK1/RIPK3 dimerization.^13^

Recent evidence suggests RIPK3 is a tumor suppressor. RIPK3 has been found to be epigenetically silenced in breast and pancreatic cancer tissue^14, 15^ and in melanoma cell line.^16^ In tumor models, loss of RIPK3 aided a TAK1-induced inflammation model of hepatocarcinogenesis,^17^ whereas loss of RIPK3 in combination with internal tandem duplication mutations of FMS-like tyrosine kinase-3 led to an increase in leukemia *in vivo,* which was because of the absence of inflammasome activation.^18^ Expression of RIPK3 in patient samples also has been associated with disease outcome. In cervical cancers, low RIPK3 protein expression in patient biopsies correlated to a reduced response to PolyIC-based adjuvant immunotherapeutic approaches.^19^ In patients suffering from intestinal bowel disease and presenting with colorectal cancer, loss of RIPK3 expression was detected in neoplastic tissue compared with non-neoplastic and the loss of expression was correlative to a poor prognosis.^20^ These findings suggest a role for RIPK3 and potentially necroptosis in tumorigenesis, as well as prognosis.

By contrast, the deletion of RIPK1, RIPK3 or MLKL in breast cancer cell lines reduced the tumorigenic potential of the cell to form colonies and subcutaneous tumors in immunocompromised mice.^21^ To add to the complexity, increased expression in RIPK3, RIPK1 and MLKL was found in patient pancreatic ductal adenocarcinoma (PDA) compared with normal tissue and the loss of RIPK3 in a V12K-Ras-induced PDA model increased survival by reducing the expression of CXCL-1/Mincle pathway and by altering the presence of tumor-suppressive immune cell.^22^ In addition, our work and others show that RIPK1/RIPK3 drive cytokine production either in conjunction with initiating necroptosis or completely independently.^23, 24^ This makes it difficult to interpret inflammation driven *in vivo* models of tumorigenesis in RIPK3-deficient mice.

We sought to differentiate the role of RIPK3 or necroptosis in tumorigenesis *versus* the tumor microenvironment by utilizing syngeneic mouse tumor models. These models can be used to simulate metastasis, what 90% of cancer patients die from rather than the primary tumor. Similar to recently published results, we found the role of RIPK3 in the tumor microenvironment was not because of the altered immune response but the ability of tumor cells to extravasate into the lung.^25^ However, we found the kinase activity of RIPK1 but not RIPK3 kinase activity was important in the extravasation step of metastasis. Loss of MLKL showed a reduction but not significant number of tumor nodules formed in the lung compared with *Ripk3*^*−/−*^. Our data shows that in addition to the possibility of activating necroptosis through a death receptor 6-mediated manner as previously found, RIPK3 has a critical role as signaling platform downstream of stimuli promoting permeability through p38/HSP27. These data support the role of RIPK1/RIPK3 as a signaling platform in promoting vascular permeability required for tumor cell extravasation.

## Results

### Loss of RIPK3 results in decreased tumor nodules in the lung

**Figure 1:**
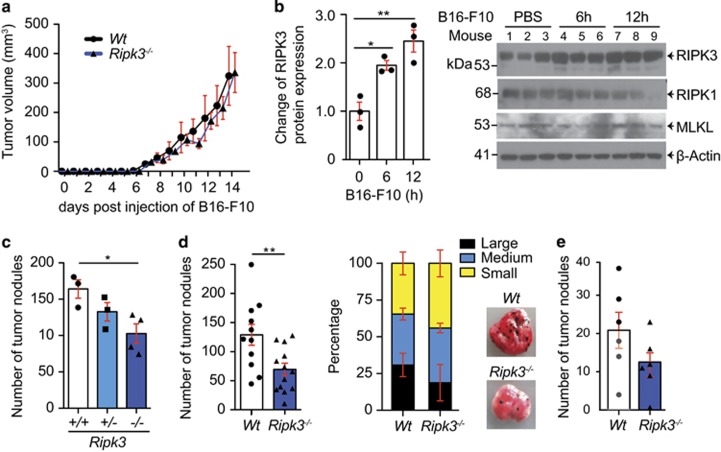
Loss of RIPK3 in the tumor microenvironment reduces tumor nodules in the lung in a tumor model of extravasation. (**a**) *Wild-type*(*Wt*) and *Ripk3*^*−/−*^ mice were injected subcutaneously with 1 × 10^5^ B16-F10 tumor cells and tumor size was measured over time (pooled data of *n*=2 experiments; 5–7 mice used per group in each experiment). (**b**) RIPK3 but not RIPK1 or MLKL protein levels were elevated in total lung lysates of wild-type mice 6 and 12 h after injection of B16-F10 cells shown by representative immunoblot and corresponding quantification of pixel density from three mice per timepoint. Fold change relative to untreated *Wt* control is shown from values normalized to actin (statistical analysis by one-way ANOVA). (**c**) Tumor nodules were counted 14 days post tail vein injection (dpi) of B16-F10 cells (2 × 10^5^) into *Ripk3* littermate control mice (left panel, *n*=1; 3–4 mice used per group) and tumor nodules were counted 14 dpi of B16-F10 cells (2 × 10^5^) into *Wt* and *Ripk3*^*−/−*^ mice (right panel, pooled data shown of *n*=3 experiments; 3–5 mice used per group in each experiment). (**d**) Corresponding size is shown of tumor nodules classified macroscopically into large (>1 mm), medium (0.5 mm–1 mm) and small (<0.5 mm) shown (pooled, *n*=3), and corresponding representative macroscopic pictures of the lungs were shown. (**e**) Tumor nodules were counted 20 dpi of MC-38 cells (3 × 10^5^) into *Wt* and *Ripk3*^*−/−*^mice (*n*=1; 6 mice used per group; *P*=0.14). Each dot represents a mouse except stated otherwise; statistical analysis by *t*-test except stated otherwise. *P<0.05, **P<0.01

To determine whether tumor cells were able to form a solid tumor, *Ripk3*^*−/−*^ mice were injected subcutaneously with B16-F10 cells. In agreement with recently published data,^22^ solid tumors formed at the same rate and volume in both wild-type and *Ripk3*^*−/−*^ mice (Figure 1a). This suggested the loss of RIPK3 in the tumor microenvironment did not affect established tumor cells from growing. To determine if RIPK3 in the tumor microenvironment could have a role in a tumor metastasis model, B16-F10 tumor cells were injected tail vein into wild-type mice. Lung homogenates were made 2, 6 and 12 h after tail vein injection and RIPK3 levels were assayed by immunoblotting. After 12 h, the level of RIPK3 protein was approximately twofold higher (Figure 1b), suggesting a potential role for RIPK3 in tumor formation in the lung. Interestingly, no changes in RIPK1 or MLKL levels were seen (Figure 1b). Using littermates, mice were injected tail vein with B16-F10 cells and 14 days post injection (dpi) lungs were counted for tumor nodules. The number of tumor nodules were reduced significantly by approximately 50% (Figures 1c; *P*<0.05). Interestingly, *Ripk3*^*+/−*^ mice showed an intermediate phenotype between wild-type and *Ripk3*^*−/−*^. Additional wild-type and *Ripk3*^*−/−*^ mice were then injected tail vein with B16-F10 cells and 14 dpi, after blinding, the lungs were counted for tumor nodules. There was a 46% (±7.6%) decrease in tumor nodules in the RIPK3 null lungs compared with wild-type (Figures 1d; *P*<0.01). The size of the tumor nodules was categorized into large, medium or small based on width of the nodule from pictures of the lungs and although not significant, there was a tendency toward smaller nodules in *Ripk3*^*−/−*^ mice (Figure 1e). To ensure the phenotype was not restricted to B16-F10 tumor model, MC-38 colon carcinoma cells were injected intravenously and tumor nodules in the lung were counted 20 dpi. Consistent with the B16-F10 tumor model, a decreased number of nodules in the *Ripk3*^*−/−*^ mice were found compared with wild-type mice (Figures 1d; *P*=0.14) suggesting the decrease in tumor nodules in the *Ripk3*^*−/−*^ was not dependent on the tumor cell type.

### Loss of RIPK1 kinase activity but not RIPK3 kinase activity reduce tumor nodule load in the lung

**Figure 2:**
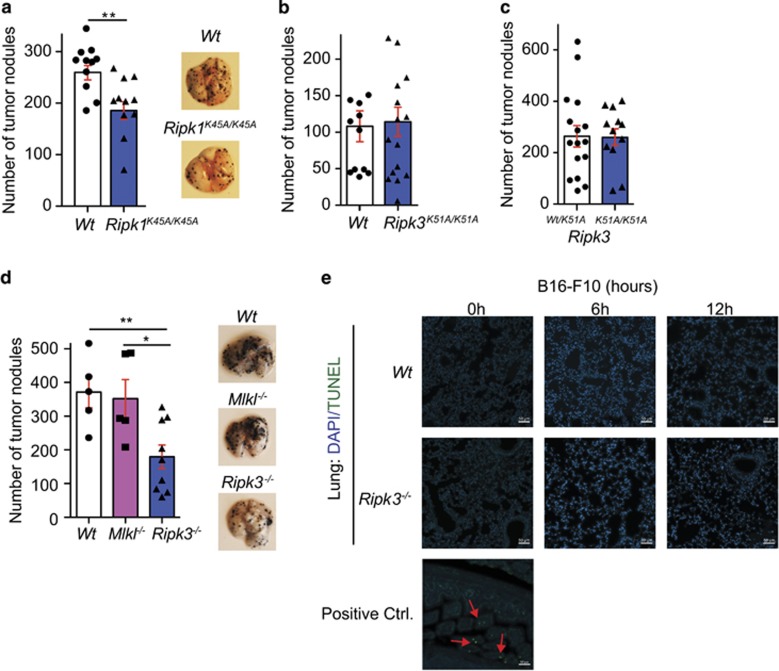
RIPK1 kinase activity but not RIPK3 kinase activity or MLKL in the tumor microenvironment is needed for tumor nodule formation in the lung. Tumor nodules were counted 14 dpi of B16-F10 cells into (**a**) *Wt* and *Ripk1*^*K45A/K45A*^ mice (pooled data of *n*=2 experiments; 4–7 mice were used per group in each experiment) and representative pictures of lungs are shown, (**b**) *Wt* and *Ripk3*^*K51A/K51A*^ mice (pooled data of *n*=2 experiments; 5–8 mice were used per group in each experiment) and (**c**) into *Ripk3*^*Wt/K51A*^ and *Ripk3*^*K51A/K51A*^ mice (pooled data of *n*=4 experiments; 3–4 mice were used per group in each experiment). (**d**) Tumor nodules were counted 14 dpi of B16-F10 cells into *Wt*, *Mlkl*^*−/−*^ and *Ripk3*^*−/−*^ mice and representative pictures are shown (data from of *n*=2 experiments; 2–6 mice were used per group in each experiment). (**e**) Paraffin-embedded lung sections from mice injected tail vein with B16-F10 at indicated timepoints were stained for TUNEL (FITC) and DAPI. Intestine of *ciap1*^*−/−*^ mice injected with TNF was used as a positive control and red arrows indicate TUNEL-positive cells. Each dot represents a mouse; statistical analysis by *t*-test; all scale bars, 50 *μ*m. **P<0.01

The necrosome is composed of RIPK1 and RIPK3 and the kinase activity of RIPK1 and RIPK3 is believed to be important and/or essential for activating necroptosis by phosphorylating MLKL. Currently, MLKL is considered to be the essential and the only known effector protein for necroptosis.^10, 11^ To determine if proteins composing the necrosome were important in the reduction of tumor nodules in the lung, RIPK1 kinase dead (K45A) mice were injected with B16-F10 cells (tail i.v.) and after 14 dpi, the lungs were harvested, blinded and tumor nodules were counted. Tumor nodules in the *Ripk1*^*K45A/K45A*^ mice were decreased by 38.3% (±8.5%) compared to wild-type mice (Figures 2a; *P*<0.05). Next, RIPK3 kinase dead (K51A) mice were injected tail vein with B16-F10 cells and again, after 14 dpi, lungs were blinded and nodules were counted in the lung. No difference in the number of tumor nodules were found (Figure 2b). As protein levels of the kinase dead RIPK3 K51A mutant have been shown to be decreased because of protein instability, we assessed the levels of protein present in lung homogenates and found the expression of the RIPK3 mutant to be significantly decreased (Supplementary Figure 1a).^26^ To control for the decreased change in expression, we compared the *Ripk3*^*wt/K51A*^ with the *Ripk3*^*K51A/K51A*^ mice. No difference in the number of tumor nodules were formed in the lung (Figure 2c). To verify that loss of RIPK3 kinase activity was able to block necroptosis, isolated endothelial cells (ECs) from the lungs of *Ripk3*^*K51A/K51A*^ mice were tested with typical activators of necroptosis, TNF, zVAD and/or Smac mimetics (Supplementary Figure 1b). As expected, compared with wild-type the RIPK3-deficient and *Ripk3*^*K51A/K51A*^-expressing cells were resistant to necroptosis caused by the combination of TNF, zVAD and Smac mimetic treatment. Interestingly, although the kinase activity of RIPK1 was required, it appeared the loss of kinase activity from RIPK3 was not required in tumor formation in the lung. Finally, to assess if necroptosis was indeed the reason for the decrease in tumor nodules in the lung, we injected B16-F10 cells tail vein into wild-type, *Mlkl*^*−/−*^ and *Ripk3*^*−/−*^ mice. Only a slight decrease in the number of tumor nodules was seen in *Mlkl*^*−/−*^ mice, whereas the reduction of tumor nodules was seen again in *Ripk3*^*−/−*^ mice (Figure 2d). The combined results from the kinase dead RIPK3 and MLKL-deficient mice suggested an alternative role for RIPK3 involving the kinase activity of RIPK1.

As the loss of RIPK3 in Vucur *et al.*^17^ led to an increase in caspase-8-dependent apoptosis in a TAK1 inflammatory-driven hepatocarcinoma, we assessed for the presence of cell death in the lung after B16-F10 cells were injected in the mice. TUNEL staining was used to determine cell death, as this technique will pick up both apoptosis or necrosis/necroptosis.^27^ For this, lung sections of mice injected with B16-F10 at different timepoints (0, 6 and 12 h) were analyzed for TUNEL-positive cells. The positive control intestinal epithelial cells from wild-type mice injected with TNF were stained positive for TUNEL, whereas lungs from wild-type or Ripk3^*−/−*^ mice did not stain TUNEL positive (Figure 2e). In addition, little to no caspase-3 activity was measured in lung homogenates of mice injected with B16-F10 at different timepoints (Supplementary Figure 1c). This suggests the kinase activity of RIPK3 and therefore the ability for necroptosis to occur in the tumor microenvironment did not alter tumor nodule formation.

### Tumor cells home to the lung in both wild-type and *Ripk3*^*−/−*^ mice with similar immune cell infiltrates

**Figure 3:**
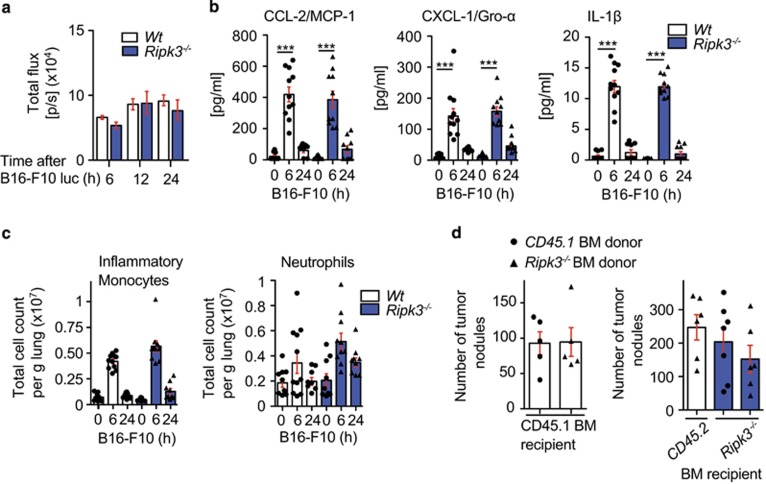
Loss of RIPK3 in the microenvironment does not alter early immune and cytokine response upon B16-F10 injection and bone marrow reconstitution shows the stromal compartment to be responsible. (**a**) B16-F10 luciferase cells home to the lung in both *Wt* and *Ripk3*^*−/−*^ mice measured by IVIS luminescent imaging (representative shown, 4–5 mice per group, *n*=2). (**b**) Cytokine levels in total lung lysates were measured by multiplex bead assay and levels of CCL-2, CXCL-1, IL-1*β* and TWEAK are shown (*n*=2). (**c**) Corresponding immune cell infiltration analysis by flow cytometry shows inflammatory monocytes (SiglecF^−^ Ly6G^−^Ly6C^hi^CCR2^+^) and neutrophil granulocyte (SiglecF^−^CD11b^+^Ly6G^+^) populations. Populations were pre-gated on singlets, live cells, Ter119^−^ and CD45^+^ cells. (**d**) Tumor nodules were counted 14 dpi of B16-F10 into CD45.1 recipient mice reconstituted with CD45.2 *Wt* and CD45.2 *Ripk3*^*−/−*^ bone marrow cells (*n*=3) and CD45.2 or *Ripk3*^*−/−*^ recipient mice reconstituted with CD45.1 or *Ripk3*^*−/−*^ bone marrow cells (*n*=2). Each dot represents a mouse; statistical analysis done by one-way ANOVA and Bonferroni post-test. ***P<0.001

To determine the additional role RIPK3 may have in metastasis, we next assessed whether the B16-F10 cells were able to home to the lung in the *Ripk3*^*−/−*^ mice. B16-F10 luciferase cells were injected and monitored after injection for movement to the lung over time. Equivalent luminescence was measured between wild-type and *Ripk3*^*−/−*^ mice within the first 24 h (Figure 3a). This suggested the B16-F10 cells were surviving and homing to the lung the same in the *Ripk3*^*−/−*^ mice compared with wild-type.

Chemokines such as C-C motif chemokine ligand 2 (CCL-2) have been shown to induce signaling to recruit inflammatory monocytes, as well as induce endothelial extravasation.^28, 29^ To determine if RIPK3 was critical in producing pro-inflammatory mediators to recruit myeloid infiltrates post injection of B16-F10 cells, cytokines/chemokines were analyzed in lungs of wild-type and *Ripk3*^*−/−*^ mice. We examined whether chemokines/cytokines were altered in *Ripk3*^*−/−*^ mice compared with wild-type mice after B16-F10 injection using lung homogenates. An increase in CCL-2, CXCL-1 and IL-1*β* was seen in both wild-type and *Ripk3*^*−/−*^ mice (Figure 3b). TNF was below the limit of detection. This suggests the ability to produce cytokines to attract monocytes was functional in the lung of *Ripk3*^*−/−*^ mice. We then assessed the immune infiltrates in the lung by flow cytometry. After 6 h post B16-F10 injection, a significant increase in the number of inflammatory monocytes (Ly6C^hi^CCR2^+^) were seen in *Ripk3*^*−/−*^ mice compared with wild-type (Figure 3c). Similarly, CD11b^+^ dendritic cells increased, whereas the CD103^+^ dendritic cell population decreased in wild-type and *Ripk3*^*−/−*^ lungs after B16-F10 injection (Supplementary Figure 2). Natural killer T cells also responded in a similar manner in both wild-type and *Ripk3*^*−/−*^ lungs after B16-F10 injection (Supplementary Figure 2).

### Loss of RIPK3 in the stroma affects tumor nodule formation in the lung

To determine the potential for the hematopoietic or stromal compartment to have a role in the reduction in tumor nodules found in *Ripk3*^*−/−*^ lungs, we performed bone marrow chimeric experiments. CD45.1 recipient mice were irradiated and reconstituted with either wild-type or RIPK3-deficient bone marrow. Only mice with reconstitution efficiency >95% were used for B16-F10 injection (data not shown). After 14 dpi, tumor nodules in the mice with RIPK3-deficient hematopoietic compartment were similar in number compared with mice with wild-type hematopoietic compartment (Figure 3d, left panel). This suggests that the hematopoietic compartment does not influence the total number of tumor nodules in the lung. We then irradiated *Ripk3*^*−/−*^ and CD45.2 mice and reconstituted the bone marrow with wild-type or RIPK3-deficient bone marrow. After 14 dpi, tumor nodules were counted. In comparison with wild-type mice reconstituted with *Ripk3*^*−/−*^ bone marrow, there was a decreasing amount of tumor nodules in the *Ripk3*^*−/−*^ mice reconstituted with wild-type (17.6%±23) or *Ripk3*^*−/−*^ bone marrow (38.6%±22.6), respectively (Figure 3d, right panel). Taken together, the data suggested that the reduction in tumor nodules in the *Ripk3*^*−/−*^ mice was because of the stromal compartment and not because of the hematopoietic compartment.

### RIPK3 promotes tumor cell extravasation independent of its kinase activity

**Figure 4:**
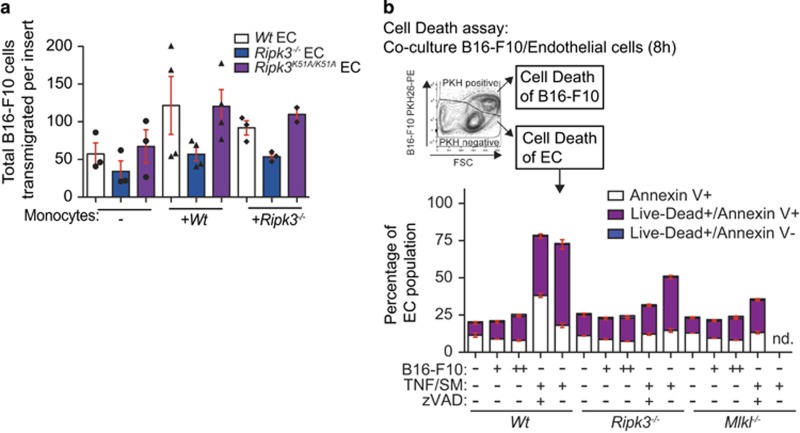
RIPK3 promotes tumor cell transendothelial migration independent of its ability to block necroptosis. (**a**) Boyden chamber transendothelial migration assay using B16-F10 cells and primary CD31^+^ ECs isolated from lungs of *Wt*, *Ripk3*^*−/−*^ or *Ripk3*^*K51A/K51A*^ mice. Primary monocytes were isolated on the day of seeding B16-F10 cells and 20 h later, transwells were fixed, stained and imaged. Image analysis was blinded and tumor cells were counted per field of view. The data are pooled from *n*=2 experiments; 1–3 transwell inserts were assayed per group in each experiment and 10–17 fields per view were analyzed per transwell insert. Total number of B16-F10 transmigrated cells per transwell insert is shown. Each dot represents an insert. Statistical analysis by one-way ANOVA and Bonferroni post-test. (**b**) EC monolayers from *Wt*, *Ripk3*^*−/−*^ and *Mlkl*^*−/−*^ mice were co-incubated with PKH26^+^ B16-F10 (+=3 × 10^4^; ++=9 × 10^4^) cells and assayed for cell death 8 h later by flow cytometry (*n*=3; nd= not determined). Cells were stained for annexin V and PI (live/dead) to identify early apoptotic cells (Live/Dead^−^ annexin V^+^) and dead cells (annexin V^+^ Live/Dead^+^ annexin V^+^ or Live/Dead^+^ annexin V^−^). Statistical analysis done by one-way ANOVA and Bonferroni post-test

To form tumors in the lung, the tumor cells must extravasate past the vascular endothelial barrier. To determine if there was a tumor cell transmigration defect, we isolated CD31^+^ cells from the lungs of wild-type, *Ripk3*^*−/−*^ or *Ripk3*^*K51A/K51A*^ mice and performed transmigration assays. A decreased number of B16-F10 cells transmigrated through *Ripk3*^*−/−*^ ECs compared with wild-type or *Ripk3*^*K51A/K51A*^ ECs (Figure 4a). We also determined if wild-type monocytes could facilitate increased tumor cell transmigration. Twofold more B16-F10 cells transmigrated through wild-type endothelial monolayer in the presence of monocytes (Figure 4a). Strikingly, neither the addition of *wild-type nor Ripk3*^*−/−*^ monocytes increased the ability of B16-F10 cells to transmigrate through a *Ripk3*^*−/−*^endothelial monolayer. By contrast, the addition of *Ripk3*^*−/−*^ monocytes did enhance B16-F10 transmigration through wild-type endothelial monolayer. Moreover, the *Ripk3*^*K51A/K51A*^ ECs allowed B16-F10 cells to transmigrate through similar to wild-type with either wild-type or *Ripk3*^*−/−*^ monocytes. These results support the idea that the hematopoietic compartment does not have a role in the loss of tumor formation in the *Ripk3*^*−/−*^ mice similar to findings in Figure 3d and further supports observation that the kinase activity of RIPK3 in the ECs is not required for the ability of tumor cells transmigrate and tumor nodules to form in the lung (Figures 2b and c).

### Cell death in primary ECs upon co-incubation of tumor cells is independent of RIPK3 or MLKL loss

To determine if necroptosis occurred to allow for transmigration, we isolated CD31^+^ cells from the lungs of wild-type, *Ripk3*^*−/−*^ and *Mlkl*^*−/−*^ mice. Primary lung EC monolayers were incubated with either 3 × 10^4^ or 9 × 10^4^ B16-F10 cells. Cell death was assessed 8 h later by flow cytometry as our *in vivo* imaging suggested the tumor cells homed to the lung within 6 h (Figure 4b). B16-F10 cells were stained for identification with the membrane dye PKH26 and were removed from the population for cell death assessment of ECs. Cells were then stained using Annexin V FITC and propidium iodide (PI) to discriminate live, early apoptotic, or late apoptotic or necrotic cells. The overall percentage of dead wild-type ECs (either early apoptotic or late apoptotic/necrotic cells) increased by 1–3% with the increasing amount of tumor cells, whereas *Ripk3*^*−/−*^ or *Mlkl*^*−/−*^ ECs only showed an increase in 1–2% dead cells upon 9 × 10^4^ tumor cell addition. However, the percentage of late apoptotic or necrotic ECs increased upon tumor cell addition was independent of the genotype of the ECs. This suggested RIPK3 may have an additional role to that of the proposed tumor cell induced necroptosis as proposed.^25^

### Loss of RIPK1 kinase activity or complete loss of RIPK3 alters permeability and vessel sprouting in response to VEGF-A

**Figure 5:**
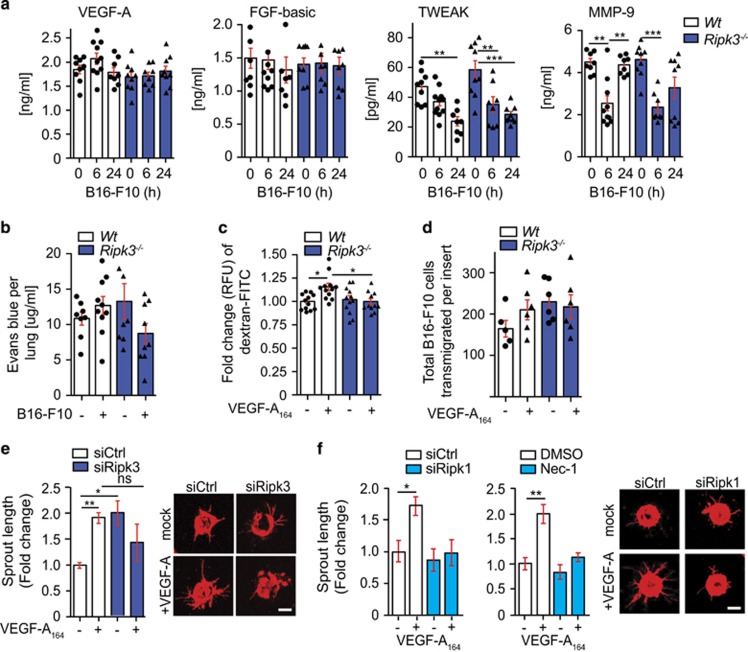
RIPK1 kinase activity and RIPK3 promotes VEGF-A-dependent vascular permeability and sprouting. (**a**) Levels of angiogenic- and remodeling factors in total lung lysates were measured by multiplex bead assay and levels of VEGF-A, FGF-basic, TWEAK and MMP-9 are shown (data pooled of *n*=2 experiment; S.E.M.; 4–6 mice were used per group; each dot represents a mouse). (**b**) Evans blue blood vessel permeability assay of Wt and *Ripk3*^*−/−*^ lungs 20 h after B16-F10 tumor cell injection i.v. (pooled data of *n*=2 experiments, 4–5 mice were used per group in each experiment; each dot represents a mouse). (**c**) Primary lung endothelial monolayer on transwell inserts were treated with VEGF-A_164_ (100 ng/ml) for 4 h and dextran-FITC permeability assay was performed. Data show relative fluorescence unit (RFU) of dextran-FITC that passed EC barrier normalized to untreated wild-type (pooled data of *n*=5 experiments; S.E.M.; 2–3 transwell inserts were used per group in each experiment; each dot represents a transwell insert). (**d**) Subsequent B16-F10 transendothelial migration assay was performed after dextran-FITC permeability assay and 20 h after B16-F10 (PKH26^+^) cells were seeded into inserts, transwells were fixed, stained and imaged. Image analysis was blinded and tumor cells were counted per field of view. The data are pooled from *n*=2 experiments; 2–3 transwell inserts were assayed per group in each experiment and 10–17 fields per view were analyzed per transwell insert. S.E.M. and total number of B16-F10 transmigrated cells per transwell insert is shown. Each dot represents an insert. Statistical analysis by one-way ANOVA and Bonferroni post-test. (**e**) HUVECs were transfected with siRNAs targeting RIPK3 or (**f**) RIPK1 (left panel) or treatment with necrostatin-1 (Nec-1, right panel) and sprouting assay was performed. Representative fluorescnece microscopy images are shown. Data from *n*=3 experiments; S.E.M.; statistical analysis by *t*-test; all scale bars, 100 *μ*m. *P<0.05, **P<0.01, ***P<0.001

Vascular permeability has been proposed as a model for tumor metastasis where vascular endothelial growth factor (VEGF), fibroblast growth factor (FGF) or CCL-2 may influence permeability.^30, 31, 32^ Recent data show that activation of VEGF leads to c-Src activation and change in VE-cadherin distribution allowing for increased vascular permeability and subsequent tumor metastasis.^33^ To determine if factors involved in vascular permeability was affected, we assayed for VEGF-A, FGF-b or TWEAK in lung homogenates of wild-type and *Ripk3*^*−/−*^ mice injected with B16-F10 cells. Interestingly, the loss of RIPK3 showed no increase in VEGF-A at 6 h after B16-F10 cell injection compared with wild-type (Figure 5a). A decrease in metalloproteinase 9 (MMP-9) was detected at 24 h after B16-F10 cell injection in the lungs of *Ripk3*^*−/−*^ compared with wild-type (Figure 5a). VEGF-A and FGF-b was also detected in supernatant of transmigration assays of either wild-type or *Ripk3*^*−/−*^ ECs with monocytes and tumor cells after 20 h. MMP-9 was decreased in the supernatant of transmigration assays of *Ripk3*^*−/−*^ ECs compared with wild-type (Supplementary Figure 3a). These data suggested that factors involved in vascular permeability were present with the exception of VEGF, which was reduced at 6 h in the lung of *Ripk3*^*−/−*^ after B16-F10 cell injection. We therefore assessed if pulmonary vascular permeability after tumor injection was affected in the *Ripk3*^*−/−*^ mice using the Evans blue permeability assay. Evans blue dye binds to albumin and uptake of the dye in organs is a measure of tissue permeability. Evans blue dye was injected into wild-type and *Ripk3*^*−/−*^ mice 20 h post injection of B16-F10 cells. The amount of Evans blue dye in wild-type lung homogenate increased slightly when B16-F10 cells were injected, but no increase in Evans blue dye was observed in *Ripk3*^*−/−*^ mice (Figure 5b).

To further assess the issue of vascular permeability, we treated primary ECs for 4 h with VEGF-A_164_ (referred hereafter as VEGF-A) and assayed for endothelial leakiness using dextran-FITC. Although a significant increase in approximately 15% of dextran-FITC detected after treating wild-type ECs with VEGF-A, there was no increase in permeability seen in treated *Ripk3*^*−/−*^ ECs (Figure 5c). We assessed whether the loss of RIPK1 kinase activity altered endothelial barrier permeability in response VEGF-A and found the loss of RIPK1 kinase activity led to no increase in endothelial permeability in response to VEGF-A (Supplementary Figure 3b). We then asked if pre-stimulation with VEGF-A could increase the number of B16-F10 cells migrating through an EC barrier. Although the number of B16-F10 cells increased by approximately 25% for a wild-type endothelial barrier, no increase in B16-F10 tumor cells was seen for a *Ripk3*^*−/−*^ endothelial barrier (Figure 5d). To determine if other functional roles of VEGF-A were also compromised by the lack of RIPK1 or RIPK3, we assessed vessel sprouting. Interestingly, when RIPK3 was downregulated in human umbilical vein ECs (HUVECs), these cells showed a twofold increase in basal vessel sprouting. Importantly, the addition of VEGF-A induced the outgrowth of vessel sprouts in wild-type HUVECs (siRNA control) but not in HUVECs where RIPK3 was silenced by siRNA (Figure 5e). Consistent with previous reports, HUVECs did not express RIPK3 protein at high levels and even a slight reduction in RIPK3 levels led to a difference in VEGF-A response in vessel sprouting (Supplementary Figure 3c). We then assessed the role of RIPK1 in vessel sprouting and similarly found that the loss of RIPK1 by siRNA or loss of kinase activity of RIPK1 by use of Nec-1 led to a decrease in vessel sprouting in response to VEGF-A compared with wild-type HUVECs (Figure 5f). Taken together, these results show that RIPK1 or RIPK3-deficient ECs fail to respond to tumor-induced vascular permeability or VEGF-A-induced permeability and angiogenesis, resulting in a defect in B16-F10 transendothelial migration.

### Loss of RIPK1 kinase activity and complete loss of RIPK3 in ECs dampens p38/HSP27 signaling in response to permeability factors

**Figure 6:**
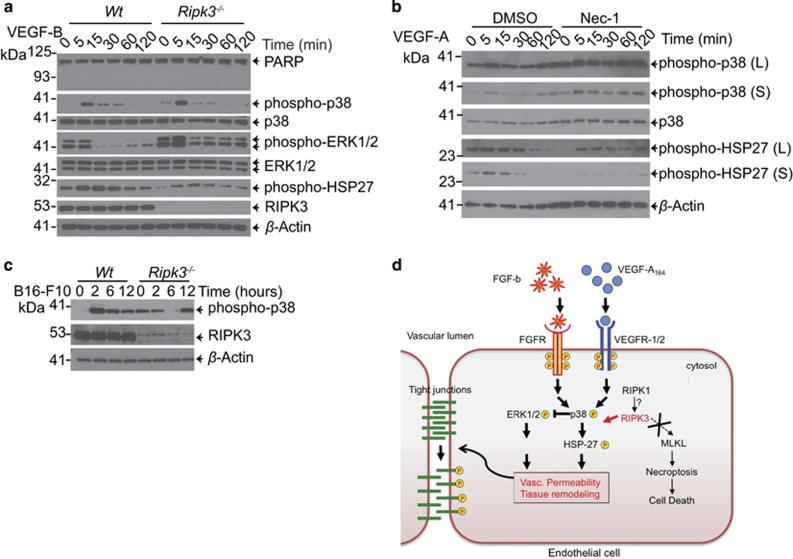
RIPK1 kinase activity and RIPK3 promotes VEGF-dependent activation of p38/HSP27 MAP kinase signaling axis. (**a**) SV40 large T immortalized ECs isolated from *Wt* or *Ripk3*^*−/−*^ mice were treated with VEGF-B (10 ng/ml) and assayed for signaling by immunoblot analysis as indicated. Representative immunoblot is shown of *n*=3 experiments. (**b**) SV40 large T immortalized ECs isolated from *Wt* mice were treated with DMSO or necrostatin-1 (Nec-1) 1 h before VEGF-A (10 ng/ml) treatment and assayed for signaling by immunoblot analysis as indicated. (**c**) Tumor cells were injected into mice at indicated timepoints and lungs were lysed and immunoblotted as indicated. (S=short exposure, L=long exposure). (**d**) Proposed model: RIPK1 kinase activity and RIPK3 promote phosphorylation of HSP27 in ECs upon permeability factor stimulation

To determine the potential mechanism of how RIPK3 may affect vascular permeability, we examined downstream signaling in response to VEGF-A, VEGF-B or FGF-b. In response to VEGF-A, VEGF-B or FGF-b, wild-type ECs showed an increase in phospho-p38 followed by the downstream phosphorylation of HSP27 protein, whereas phospho-ERK1/2 decreased in signal (Figure 6a and Supplementary Figure 4a). The inverse status of phospho-p38 and phospho-ERK1/2 in response to VEGF-A has been previously shown in HUVECs where activation of p38 leads to decreased levels of ERK1/2 activation.^34^ By contrast, RIPK3 null ECs stimulated with either VEGF-A, VEGF-B or FGF-b resulted in reduced phospho-p38 activation compared with wild-type. More strikingly, the downstream phosphorylated protein, HSP27, was further reduced (Figure 6a and Supplementary Figure 4a). Conversely, phosphorylated ERK1/2 in the *Ripk3*^*−/−*^ ECs was increased compared with wild-type reflecting the dampened p38 activation in the RIPK3 knockouts. Interestingly, *Ripk3*^*−/−*^ ECs without stimulation, had an increased basal level of phospho-p38 present compared with wild-type cells. No major differences in the activation of p38/HSP27 between wild-type and *Ripk3*^*−/−*^ ECs was seen when treated with TNF (Supplementary Figure 4a and 4b). The inhibition of RIPK1 activity by necrostatin-1 reduced HSP27 phosphorylation similarly to the loss of RIPK3 when ECs were treated with VEGF-A (Figure 6b). To determine if the signaling differences occurred *in vivo* after tumor cell injection, we injected mice with tumor cells and after 2, 6 and 12 h, the lungs were homogenized for protein lysates. Consistent with our immortalized ECs, basal levels of phospho-p38 occurred in the *Ripk3*^*−/−*^ lungs compared with wild-type. At 2 h, phospho-p38 was upregulated in wild-type mice but not in *Ripk3*^*−/−*^ mice (Figure 6c). Taken together, our data show that RIPK3 positively regulates the activation of p38 and HSP27 upon permeability factor treatment (VEGF-A, VEGF-B, FGF-b) and therefore promoting permeability in the tumor cells to metastasize (Figure 6d).

## Discussion

The current dogma is that RIPK3 causes inflammation because of the induction of necroptosis. However, our previous results suggest that RIPK3 may alter cytokine release, particularly TNF in response to loss of IAPs.^24^ We therefore set out to determine if RIPK3 may have a role in the tumor microenvironment by promoting cytokine production or causing necroptosis leading to tumor progression. Our results show RIPK3 did not alter tumor growth of established tumors. In addition, changes in cytokine production in *Ripk3*^*−/−*^ mice compared with wild-type mice after tumor injection were minimal. The loss of kinase activity of RIPK1 and the complete loss of RIPK3 were found to be important in the ability of the tumor cells to form lung nodules. Surprisingly, we did not find *Ripk3*^*K51A/K51A*^ or *Mlkl*^*−/−*^ mice exhibited a reduction of tumor nodules in the lung similar to *Ripk3*^*−/−*^ mice. Bone marrow chimeras showed the involvement of the stromal but not the hematopoietic compartment was critical for the loss of tumor nodules in the lung in *Ripk3*^*−/−*^ mice. We then found the ability of tumor cells to transmigrate through *Ripk3*^*−/−*^ endothelial barrier to be deficient and that cell death in the EC layer was independent of the genotype when tumor cells were co-incubated. We unexpectedly found the loss of RIPK3 or the kinase activity of RIPK1 in the endothelial compartment were defective in p38/HSP27 signaling in response to permeability factors such as VEGF-A. This lack of response resulted in decreased permeability and vessel sprouting in response to VEGF-A. These results suggest the role of the necrosome is minimal in the extravasation process of tumor cells and presents a novel role for RIPK3 as a signaling platform downstream of angiogenic factors such as VEGF and FGF.

The current difficulty in assessing the role of necroptosis in related pathologies of human inflammatory disease is the absence of identifying markers. The use of RIPK3 genetic deletion, pharmacological inhibition of RIPK1 and/or correlative evidence of RIPK3 protein or RNA upregulation has been used to associate the disease state with necroptosis.^35, 36^ However, as RIPK1 has been shown to have alternative function in regulating apoptosis and necroptosis^37, 38, 39^ and also directly influencing inflammation,^24, 40, 41^ further downstream effectors of necroptosis such as the use of *Mlkl*^*−/−*^ mice are required to prove the role of necroptosis.^42^ In our experiments, the number of pulmonary tumor nodules in the *Mlkl*^*−/−*^ mice were slightly reduced compared with wild-type mice but not to the same extent as in the *Ripk3*^*−/−*^ mice. It is unclear what may be the reason for the difference in the tumor nodule numbers in the MLKL knock-out mice we obtained compared with Strilic *et al.*^25^, except for the source of the MLKL null mice. Our data also show that the kinase activity of RIPK1 has a role in the ability of tumor nodules to form in the lung. Activation of VEGFR2 by VEGF-A or VEGF-B causes phosphorylation of c-Src and has been linked to increased permeability by altering adherens junction formation, VE-cadherin.^33, 43^ c-Src has been shown to inhibit Fas-induced caspase-8 activation by phosphorylation at Tyr380.^44^ Further studies are required to determine if RIPK1 and RIPK3 alter tumor cell extravasation as a complex with or without caspase-8 involvement or if these proteins regulate different pathways to result in vascular permeability.

Vascular permeability requires tissue remodeling similar to wound healing. Impaired wound healing was found in *Ripk3*^*−/−*^ mice compared with wild-type mice, characterized by a decreased MMP-9 protein expression and delayed CD31^+^ staining and VEGF production.^45^ Of the cytokines and growth factors we assayed, only MMP-9 was reduced at 24 h in the *Ripk3*^*−/−*^ mice compared with wild-type upon tumor cell injection. The decrease in MMP-9 protein levels correspond to an increase in neutrophil infiltration of the lung at 24 h in the *Ripk3*^*−/−*^, suggesting an attempt of the immune system to compensate for the lack of response in the endothelial compartment. The ability of *Ripk3*^*−/−*^ monocytes to enhance transmigration of tumor cells through a wild-type endothelial layer further suggests a defect in the *Ripk3*^*−/−*^ ECs to respond to extracellular cues. Indeed, the absence of RIPK3 reduced signaling in response to VEGF-A, VEGF-B and FGF-b but not TNF in the p38/HSP27 pathway, a key pathway in permeability. Our results show that the reduced tumor nodule formation in RIPK3-deficient mice cannot be attributed to the failure to respond to one stimuli alone. Interestingly, in Seifert *et al.*^22^, the loss of Mincle combined with the V12K-Ras PDA did not protect, as well as the loss of RIPK3, suggesting additional factors may have a role in the ability of RIPK3 to form PDA in a V12K-Ras model. Alternatively, the role of RIPK3 may be more prevalent in tissues that re-model frequently or tissues that must function as a barrier for infection. Further investigation of other permeability factors *in vivo* and whether these ligands can form complexes of RIPK1/RIPK3 will be of interest to determine if these intracellular proteins can be targeted to block several pathways involved in tumor metastasis.

*Ripk3*^*−/−*^ mice have not been reported to have any vascular defects during normal development. Interestingly, we repeatedly detect increased basal phospho-p38 levels in untreated immortalized ECs and lung homogenates suggesting the loss of RIPK3 is compensated during development and regular homeostasis. It appears only during stress such as wound healing or tumor challenge, does RIPK3 become required for the response to the additional stimuli. Changes at the basal level in structure of the vessels or lung vessel structure have not been assessed and may provide insight to why the *Ripk3*^*−/−*^ mice develop with little to no overt phenotype.

In summary, our study shows the kinase activity of RIPK1 and RIPK3 has a physiological role in the tumor microenvironment, in particular tumor cell extravasation and remodeling by altering the downstream signaling pathways of permeability factors. This is a novel role for the kinase activity of RIPK1/RIPK3 in addition to its role in regulating necroptosis. In the context of inflammation and cell death, this supports previous reports suggesting the loss of kinase activity of RIPK1 or the complete loss of RIPK3 cannot be used as an indicator of necroptosis and the potential of RIPK1/RIPK3 to be a signaling platform will need to be assessed in each disease model individually.

## Materials and methods

### Animal work

Animals were maintained under optimized hygiene conditions (OHB), and experiments were approved by Zürich Cantonal Veterinary Committee in accordance to the guidelines of the Swiss Animal Protection Law (License 119/2012 and 186/2015). C57BL/6 mice were purchased from Janvier Labs (France), *Ripk3*^*−/−*^ mice were a kind gift from V Dixit,^46^ SNP analyzed at *N*=7, *Ripk1*^*K45A/K45A*^, *Ripk3*^*K51A/K51A*^ *mice* were a kind gift from John Bertin and Peter J Gough from GlaxoSmithKline (PA, USA),^26, 40^ *Mlkl*^*−/−*^ mice were obtained from J Murphy and W Alexander^10^ (WEHI, Melbourne, Australia). Mice were injected with B16-F10 (2 × 10^5^ cells) or MC-38 (3 × 10^5^ cells) intravenously in 200 *μ*l PBS or subcutaneous in 100* μ*l PBS (1 × 10^5^). Mice were killed at indicated timepoints by overdose (ketamin/xylazin cocktail), lungs perfused and blinded before further processing (nodule counting, flow cytometric analysis). For bone marrow chimeric experiments, Wt CD45.1 or *Ripk3*^*−/−*^ mice were irradiated two times at 550 rads, 6 h apart, injected with donor bone marrow and assessed for reconstitution efficiency at 6 weeks. Only mice with reconstitution efficiency >95% were used for further experiments.

### Cell culture

MC-38 and B16-F10 cells were cultured at maximum 50% confluence in Dulbecco's modified Eagle's medium (Gibco, ThemoFisher Scientific, Germany) with high glucose (4.5 g/l) supplemented with 10% fetal bovine serum (FBS; Gibco, ThemoFisher Scientific) and penicillin/streptomycin/glutamine (Gibco, ThemoFisher Scientific). Primary- and SV40 immortalized lung ECs and HUVECs (C-12200, Promocell, Germany) were cultured in EBM-2 media supplemented with EGM according to the manufacturers recommendation (Lonza, Germany).

### Primary lung EC isolation

Lungs were perfused with PBS and HBSS(+Collagenase 1 mg/ml; Sigma Aldrich, Switzerland) and minced before incubation with HBSS(+Collagenase) for 45 min and further processed for antibody incubation. Mouse lung cell suspension was incubated with anti-CD31(eBioscience, Austria, clone 390) antibody for 30 min in PBS (0.5% BSA) at 1 : 20 dilution in order to isolate lung ECs. To isolate monocytes from bone marrow cell suspension, the anti-CD115-biotin (eBioscience, clone AFS98) antibody was used at dilution of 1 : 50 in HBSS+2% FBS. Cell suspensions were incubated in primary antibody for 30 min at 4 °C followed by incubation with anti-rat IgG or streptavidin magnetic particles and sorted on LS columns according to the manufacturers recommendations (Miltenyi, Switzerland).

### Transendothelial migration assay

EC populations >85% purity for CD31^+^ cells were used. In all, 3 × 10^4^ primary ECs were seeded 5–8 days after isolation on a gelatin coated insert (Corning, Netherlands, 8 *μ*m pore size) in EBM-2 (Lonza). Two days after seeding, cell culture media was changed RPMI (Gibco) in bottom well (3% FBS) and insert (1% FBS) with or without pre-treatement of VEGF (100 *μ*g/ml) for 4 or 1 h before addition of 2 × 10^4^ PKH26^+^ (Sigma Aldrich) membrane stained B16-F10 tumor cells and/or 1 × 10^5^ primary isolated monocytes (CD115^+^). After 20 h, the remaining cells inside the insert were removed, membrane fixed in 2% PFA and mounted using slow fade gold with dapi (Thermoscience Fisher, Germany). Microscopic fluorescence picture were taken at × 10 magnification using ZEISS AXIO Scan Z.1 Centre for Microscopy and Image Analysis (ZMB), Switzerland. PKH26^+^ cells were counted per field of view. A minimum of 10–17 field of views were counted per transwell.

### Dextran-FITC permeability assay

In all, 3 × 10^4^ primary ECs were seeded 5–8 days after isolation on a gelatin-coated insert (Corning, 8 *μ*m pore size) in EBM-2 (Lonza) and grown to confluent monolayer for 3 days. Cells were then pre-treated with VEGF-A_164_ for 4 h before dextran-FITC was added into insert at final concentration of 1 mg/ml. Supernatant from bottom well was collected 1 h after addition of dextran-FITC and fluorescence intensity was measured by TECAN Infinite 200 Pro multireader (Switzerland) using *λ*_Ex_=485 nm and *λ*_Em_=525 nm. Relative fluorescence unit was quantified.

### HUVEC sprouting assay

HUVECs were transfected with Oligofectamine Reagent (Invitrogen, Germany) with 200 nM siRNA universal negative control (SIC001, Sigma Aldrich) or siRNA against RIPK1 (sense 5′-CCACUAGUCUGACGGAUAA[dT][dT]-3′) or RIPK3 (sense 5′-CCAGAGACCUCAACUUUCA[dT][dT]-3′). After 2 days, transfected cells were coated on cytodex beads and embedded on a fibrinogen gel. Cells were treated with or without VEGF (50 ng/ml) and/or Nec-1 (S8037, Abscource Diagnostics GmbH, Germany, 25 *μ*M) for 24 h. After treatment, cells were fixed (4%PFA for 30 min), permeabilized (0.2%Triton X-100 in PBS for 10 min) and stained with phalloidin (1 *μ*g/ml in PBS, P1951 Sigma Aldrich) for 1 h. Images were obtained with a Zeiss LSM 510 META confocal microscope (Germany) (10x) and quantification was done with ImageJ.

### Cytokine measurement

Cell culture supernatant and lung lysates were prepared according to the manufacturer's instructions. Multiplex cytokine kits from eBioscience/Affymetrix (Austria) and BioTechne (UK) were used. The measurements were performed on Bio-Plex 200 system (Bio-Rad Laboratories, Switzerland) and data were analyzed by Bio-Plex software 6.0.

### Immunohistochemistry

Organs were fixed in 4% paraformaldehyde, paraffin embedded and 4 *μ*m sections were stained for TUNEL according to the manufacturer recommendation (Biotool, Germany). Heat-induced epitope retrieval with citrate buffer, blocked in 3% goat serum, permeabilized with 0.3% Triton X-100 and stained with cleaved caspase-3 (Cell Signaling Technology, MA, USA) or TUNEL. Microscopic fluorescence picture were taken at indicated magnification using ZEISS AXIO Scan Z.1 (10x and 20x) microscope.

### Antibodies

Flow cytometry analysis was performed according to standard procedure and the following antibodies were purchased from eBioscience unless noted otherwise. The following antibodies were used: CD45.1-eFluor 450, CD45.2-APC-Cy7, CD45-brilliant violet 605, CD11b-PE-Cy7, Ly6C-brilliant violet 711, CD11c-brilliant violet 510, Ly6G-PerCP-Cy5.5, MHC-II-alexa fluor 700, CD103-FITC, SiglecF-brilliant violet 421, CD86-brilliant violet 650, Ter119-brilliant violet 421, CCR-2-APC (BioTechne), fixable live/dead eFluor 780 or PI for live/dead assays. For immunoblotting, antibodies were diluted at recommended dilution in 5% skim milk in PBS (0.1% Tween). The following antibodies were used: RIPK3 (BioTechne), phospho-p38, total p38, phospho-ERK1/2, total ERK1/2, phospho-HSP27 (clone D1H2) from Cell Signaling Technology, beta-actin (clone AC-15, Sigma Aldrich), HSP70 (clone 3A3 Santa Cruz Biotechnology, CA, USA), secondary anti-rat IgG and anti-mouse IgG (Southern Biotec).

### Statistics

*N* refers to the number of independent experiments performed. For each experiment, 3–8 mice were used per group. Every dot shown in the graphs represents an individual mouse. GraphPad Prism (GraphPad Software, CA, USA) was used for statistical analyses. All *P*-values were calculated by either *t*-test or one-way ANOVA as indicated in the corresponding figure legend and indicated as asterisks (*P⩽0.05, **P⩽0.01, ***P⩽0.001).

## References

[bib1] Hanahan D, Weinberg RA. Hallmarks of cancer: the next generation. Cell 2011; 144: 646–674. 21376230 10.1016/j.cell.2011.02.013

[bib2] Cho YS, Challa S, Moquin D, Genga R, Ray TD, Guildford M et al. Phosphorylation-driven assembly of the RIP1-RIP3 complex regulates programmed necrosis and virus-induced inflammation. Cell 2009; 137: 1112–1123. 19524513 10.1016/j.cell.2009.05.037 PMC2727676

[bib3] Zhang D-W, Shao J, Lin J, Zhang N, Lu B-J, Lin S-C et al. RIP3, an energy metabolism regulator that switches TNF-induced cell death from apoptosis to necrosis. Science 2009; 325: 332–336. 19498109 10.1126/science.1172308

[bib4] Kaiser WJ, Sridharan H, Huang C, Mandal P, Upton JW, Gough PJ et al. Toll-like receptor 3-mediated necrosis via TRIF, RIP3, and MLKL. J Biol Chem 2013; 288: 31268–31279. 24019532 10.1074/jbc.M113.462341 PMC3829437

[bib5] He S, Wang L, Miao L, Wang T, Du F, Zhao L et al. Receptor interacting protein kinase-3 determines cellular necrotic response to TNF-alpha. Cell 2009; 137: 1100–1111. 19524512 10.1016/j.cell.2009.05.021

[bib6] Holler N, Zaru R, Micheau O, Thome M, Attinger A, Valitutti S et al. Fas triggers an alternative, caspase-8-independent cell death pathway using the kinase RIP as effector molecule. Nat Immunol 2000; 1: 489–495. 11101870 10.1038/82732

[bib7] Ofengeim D, Yuan J. Regulation of RIP1 kinase signalling at the crossroads of inflammation and cell death. Nat Rev Mol Cell Biol 2013; 14: 727–736. 24129419 10.1038/nrm3683

[bib8] Sun L, Wang H, Wang Z, He S, Chen S, Liao D et al. Mixed lineage kinase domain-like protein mediates necrosis signaling downstream of RIP3 kinase. Cell 2012; 148: 213–227. 22265413 10.1016/j.cell.2011.11.031

[bib9] Newton K, Dugger DL, Wickliffe KE, Kapoor N, de Almagro MC, Vucic D et al. Activity of protein kinase RIPK3 determines whether cells die by necroptosis or apoptosis. Science 2014; 343: 1357–1360. 24557836 10.1126/science.1249361

[bib10] Murphy JM, Czabotar PE, Hildebrand JM, Lucet IS, Zhang J-G, Alvarez-Diaz S et al. The pseudokinase MLKL mediates necroptosis via a molecular switch mechanism. Immunity 2013; 39: 443–453. 24012422 10.1016/j.immuni.2013.06.018

[bib11] Hildebrand JM, Tanzer MC, Lucet IS, Young SN, Spall SK, Sharma P et al. Activation of the pseudokinase MLKL unleashes the four-helix bundle domain to induce membrane localization and necroptotic cell death. Proc Natl Acad Sci USA 2014; 111: 15072–15077. 25288762 10.1073/pnas.1408987111 PMC4210347

[bib12] Dondelinger Y, Declercq W, Montessuit S, Roelandt R, Goncalves A, Bruggeman I et al. MLKL compromises plasma membrane integrity by binding to phosphatidylinositol phosphates. Cell Rep 2014; 7: 1–11. 24813885 10.1016/j.celrep.2014.04.026

[bib13] Yatim N, Jusforgues-Saklani H, Orozco S, Schulz O, Barreira da Silva R, Reis e Sousa C et al. RIPK1 and NF-κB signaling in dying cells determines cross-priming of CD8^+^ T cells. Science 2015; 350: 328–334. 26405229 10.1126/science.aad0395 PMC4651449

[bib14] Koo G-B, Morgan MJ, Lee D-G, Kim W-J, Yoon J-H, Koo JS et al. Methylation-dependent loss of RIP3 expression in cancer represses programmed necrosis in response to chemotherapeutics. Cell Res 2015; 25: 707–725. 25952668 10.1038/cr.2015.56 PMC4456623

[bib15] Tan AC, Jimeno A, Lin SH, Wheelhouse J, Chan F, Solomon A et al. Characterizing DNA methylation patterns in pancreatic cancer genome. Mol Oncol 2009; 3: 425–438. 19497796 10.1016/j.molonc.2009.03.004 PMC5527529

[bib16] Geserick P, Wang J, Schilling R, Horn S, Harris PA, Bertin J et al. Absence of RIPK3 predicts necroptosis resistance in malignant melanoma. Cell Death Dis 2015; 6: e1884–12. 26355347 10.1038/cddis.2015.240 PMC4650439

[bib17] Vucur M, Reisinger F, Gautheron J, Janssen J, Roderburg C, Cardenas DV et al. RIP3 inhibits inflammatory hepatocarcinogenesis but promotes cholestasis by controlling caspase-8- and JNK-dependent compensatory cell proliferation. Cell Rep 2013; 4: 776–790. 23972991 10.1016/j.celrep.2013.07.035

[bib18] Höckendorf U, Yabal M, Herold T, Munkhbaatar E, Rott S, Jilg S et al. RIPK3 restricts myeloid leukemogenesis by promoting cell death and differentiation of leukemia initiating cells. Cancer Cell 2016; 30: 75–91. 27411587 10.1016/j.ccell.2016.06.002

[bib19] Schmidt SV, Seibert S, Walch-Rückheim B, Vicinus B, Kamionka E-M, Pahne-Zeppenfeld J et al. RIPK3 expression in cervical cancer cells is required for PolyIC-induced necroptosis, IL-1α release, and efficient paracrine dendritic cell activation. Oncotarget 2015; 6: 8635–8647. 25888634 10.18632/oncotarget.3249 PMC4496172

[bib20] Bozec D, Iuga AC, Roda G, Dahan S, Yeretssian G. Critical function of the necroptosis adaptor RIPK3 in protecting from intestinal tumorigenesis. Oncotarget 2016; 7: 46384–46400. 27344176 10.18632/oncotarget.10135 PMC5216805

[bib21] Liu X, Zhou M, Mei L, Ruan J, Hu Q, Peng J et al. Key roles of necroptotic factors in promoting tumor growth. Oncotarget 2016; 7: 22219–22233. 26959742 10.18632/oncotarget.7924 PMC5008357

[bib22] Seifert L, Werba G, Tiwari S, Ly NNG, Alothman S, Alqunaibit D et al. The necrosome promotes pancreatic oncogenesis via CXCL1 and Mincle-induced immune suppression. Nature 2016; 532: 1–17. 10.1038/nature17403 PMC4833566 27049944

[bib23] Moriwaki K, Balaji S, Mcquade T, Malhotra N, Kang J, Chan FK-M. The necroptosis adaptor RIPK3 promotes injury-induced cytokine expression and tissue repair. Immunity 2014; 41: 567–578. 25367573 10.1016/j.immuni.2014.09.016 PMC4220270

[bib24] Wong WW-L, Vince JE, Lalaoui N, Lawlor KE, Chau D, Bankovacki A et al. cIAPs and XIAP regulate myelopoiesis through cytokine production in an RIPK1- and RIPK3-dependent manner. Blood 2014; 123: 2562–2572. 24497535 10.1182/blood-2013-06-510743

[bib25] Strilic B, Yang L, Albarrán-Juárez J, Wachsmuth L, Han K, Müller UC et al. Tumour-cell-induced endothelial cell necroptosis via death receptor 6 promotes metastasis. Nature 2016; 536: 215–218. 27487218 10.1038/nature19076

[bib26] Mandal P, Berger SB, Pillay S, Moriwaki K, Huang C, Guo H et al. RIP3 induces apoptosis independent of pronecrotic kinase activity. Mol Cell 2014; 56: 481–495. 25459880 10.1016/j.molcel.2014.10.021 PMC4512186

[bib27] Graslkraupp B, Ruttkaynedecky B, Koudelka H, Bukowska K, Bursch W, Schultehermann R. *In-situ*detection of fragmented DNA (Tunel assay) fails to discriminate among apoptosis, necrosis, and autolytic cell-death - a cautionary note. Hepatology 1995; 21: 1465–1468. 7737654 10.1002/hep.1840210534

[bib28] Qian B-Z, Li J, Zhang H, Kitamura T, Zhang J, Campion LR et al. CCL2 recruits inflammatory monocytes to facilitate breast-tumour metastasis. Nature 2011; 475: 222–225. 21654748 10.1038/nature10138 PMC3208506

[bib29] Wolf MJ, Hoos A, Bauer J, Boettcher S, Knust M, Weber A et al. Endothelial CCR2 signaling induced by colon carcinoma cells enables extravasation via the JAK2-Stat5 and p38MAPK pathway. Cancer Cell 2012; 22: 91–105. 22789541 10.1016/j.ccr.2012.05.023

[bib30] García-Román J, Zentella-Dehesa A. Vascular permeability changes involved in tumor metastasis. Cancer Lett 2013; 335: 259–269. 23499893 10.1016/j.canlet.2013.03.005

[bib31] Carmeliet P, Lampugnani MG, Moons L, Breviario F, Compernolle V, Bono F et al. Targeted deficiency or cytosolic truncation of the VE-cadherin gene in mice impairs VEGF-mediated endothelial survival and angiogenesis. Cell 1999; 98: 147–157. 10428027 10.1016/s0092-8674(00)81010-7

[bib32] Murakami M, Nguyen LT, Zhuang ZW, Zhang ZW, Moodie KL, Carmeliet P et al. The FGF system has a key role in regulating vascular integrity. J Clin Invest 2008; 118: 3355–3366. 18776942 10.1172/JCI35298 PMC2528913

[bib33] Li X, Padhan N, Sjöström EO, Roche FP, Testini C, Honkura N et al. VEGFR2 pY949 signalling regulates adherens junction integrity and metastatic spread. Nat Commun 2016; 7: 11017. 27005951 10.1038/ncomms11017 PMC4814575

[bib34] McMullen ME, Bryant PW, Glembotski CC, Vincent PA, Pumiglia KM. Activation of p38 has opposing effects on the proliferation and migration of endothelial cells. J Biol Chem 2005; 280: 20995–21003. 15790570 10.1074/jbc.M407060200

[bib35] Luedde M, Lutz M, Carter N, Sosna J, Jacoby C, Vucur M et al. RIP3, a kinase promoting necroptotic cell death, mediates adverse remodelling after myocardial infarction. Cardiovasc Res 2014; 103: 206–216. 24920296 10.1093/cvr/cvu146

[bib36] Pierdomenico M, Negroni A, Stronati L, Vitali R, Prete E, Bertin J et al. Necroptosis is active in children with inflammatory bowel disease and contributes to heighten intestinal inflammation. Am J Gastroenterol 2013; 109: 279–287. 24322838 10.1038/ajg.2013.403

[bib37] Rickard JA, O'Donnell JA, Evans JM, Lalaoui N, Poh AR, Rogers T et al. RIPK1 regulates RIPK3-MLKL-driven systemic inflammation and emergency hematopoiesis. Cell 2014; 157: 1175–1188. 24813849 10.1016/j.cell.2014.04.019

[bib38] Dillon CP, Weinlich R, Rodriguez DA, Cripps JG, Quarato G, Gurung P et al. RIPK1 blocks early postnatal lethality mediated by caspase-8 and RIPK3. Cell 2014; 157: 1189–1202. 24813850 10.1016/j.cell.2014.04.018 PMC4068710

[bib39] Kaiser WJ, Daley-Bauer LP, Thapa RJ, Mandal P, Berger SB, Huang C et al. RIP1 suppresses innate immune necrotic as well as apoptotic cell death during mammalian parturition. Proc Natl Acad Sci USA 2014; 111: 7753–7758. 24821786 10.1073/pnas.1401857111 PMC4040608

[bib40] Berger SB, Kasparcova V, Hoffman S, Swift B, Dare L, Schaeffer M et al. Cutting edge: RIP1 kinase activity is dispensable for normal development but is a key regulator of inflammation in SHARPIN-deficient mice. J Immunol 2014; 192: 5476–5480. 24821972 10.4049/jimmunol.1400499 PMC4048763

[bib41] Shutinoski B, Alturki NA, Rijal D, Bertin J, Gough PJ, Schlossmacher MG et al. K45A mutation of RIPK1 results in poor necroptosis and cytokine signaling in macrophages, which impacts inflammatory responses *in vivo*. Cell Death Differ 2016; 23: 1–10. 27258786 10.1038/cdd.2016.51 PMC5041191

[bib42] Newton K, Dugger DL, Maltzman A, Greve JM, Hedehus M, Martin-McNulty B et al. RIPK3 deficiency or catalytically inactive RIPK1 provides greater benefit than MLKL deficiency in mouse models of inflammation and tissue injury. Cell Death Differ 2016; 23: 1–12. 27177019 10.1038/cdd.2016.46 PMC5072432

[bib43] Sun Z, Li X, Massena S, Kutschera S, Padhan N, Gualandi L et al. VEGFR2 induces c-Src signaling and vascular permeability *in vivo* via the adaptor protein TSAd. J Exp Med 2012; 209: 1363–1377. 22689825 10.1084/jem.20111343 PMC3405501

[bib44] Cursi S, Rufini A, Stagni V, Condò I, Matafora V, Bachi A et al. Src kinase phosphorylates caspase-8 on Tyr380: a novel mechanism of apoptosis suppression. EMBO J 2006; 25: 1895–1905. 16619028 10.1038/sj.emboj.7601085 PMC1456929

[bib45] Godwin A, Sharma A, Yang W-L, Wang Z, Nicastro J, Coppa GF et al. Receptor-interacting protein kinase 3 deficiency delays cutaneous wound healing. PLoS ONE 2015; 10: e0140514. 26451737 10.1371/journal.pone.0140514 PMC4599740

[bib46] Newton K, Sun X, Dixit VM. Kinase RIP3 is dispensable for normal NF-kappa Bs, signaling by the B-cell and T-cell receptors, tumor necrosis factor receptor 1, and Toll-like receptors 2 and 4. Mol Cell Biol 2004; 24: 1464–1469. 14749364 10.1128/MCB.24.4.1464-1469.2004 PMC344190

