# Supplementary material for: RIPK1/RIPK3 promotes vascular permeability to allow tumor cell extravasation independent of its necroptotic function
Source: Cell Death Dis. 2017 Feb 2;8(2):e2588–. doi: 10.1038/cddis.2017.20 (PMC5386469; doi:10.1038/cddis.2017.20)
Supplement: Supplementary Figure 1 [file cddis201720x1.pdf]

## Supplementary Figure 1

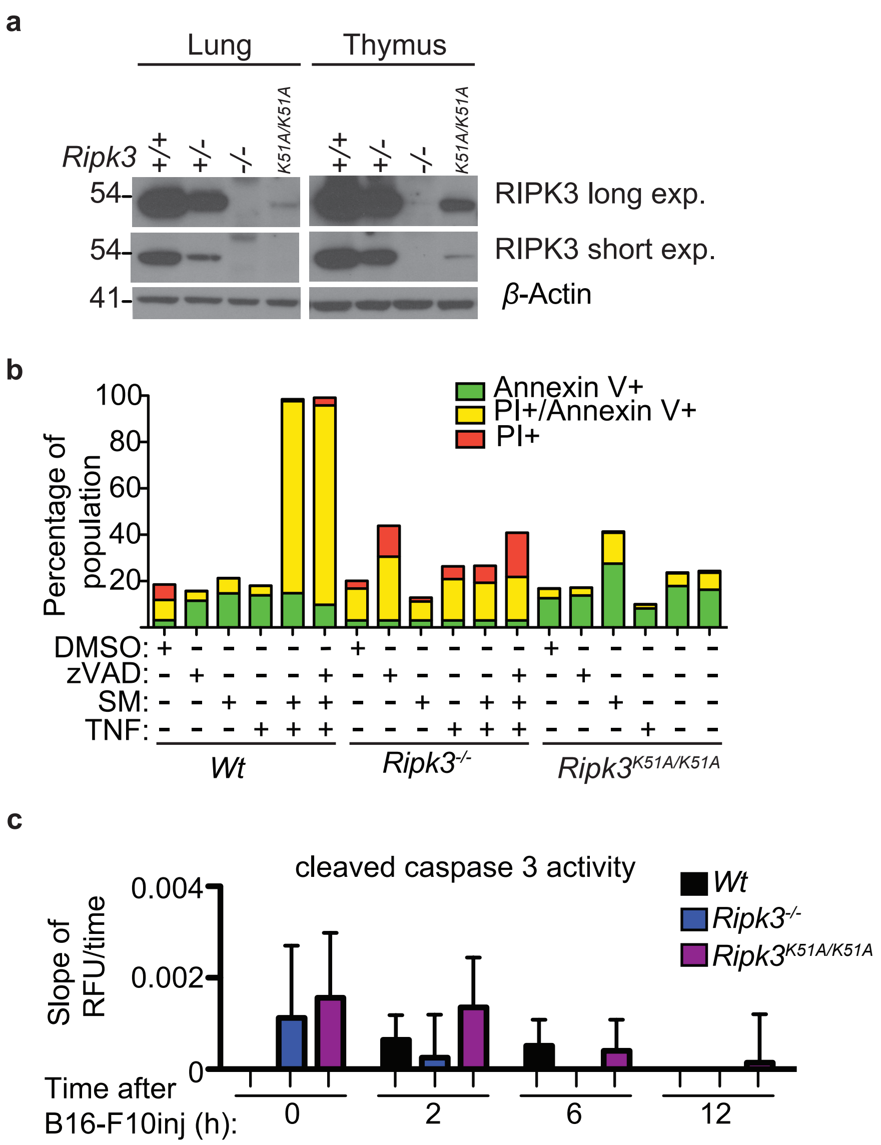


**Supplementary Figure 1. (a)** RIPK3 protein expression in lung and thymus from *Wt, Ripk3*$^{+/-}$, *Ripk3*$^{-/-}$, *Ripk3*$^{K51A/K51A}$ mice. **(b)** Cell death was measured using flow cytometry analyis by staining live/dead cells with propidium iodide (PI) and apoptotic cells by (Annexin-V FITC) after treatment of endothelial cells of *Wt, Ripk3*$^{-/-}$ *and Ripk3*$^{K51A/K51A}$ mice for 24h with indicated combinations of TNF (100ng/ml), smac-mimetic compound A (SM, 500nM) or zVAD-FMK (5ug/ml). Quantification of one representative experiment shown (n=3).**(c)** Cleaved caspase 3 activity assay from lung lysates of mice injected with B16-F10 (3-4 mice per condition).
