# Supplementary material for: RIPK1/RIPK3 promotes vascular permeability to allow tumor cell extravasation independent of its necroptotic function
Source: Cell Death Dis. 2017 Feb 2;8(2):e2588–. doi: 10.1038/cddis.2017.20 (PMC5386469; doi:10.1038/cddis.2017.20)
Supplement: Supplementary Figure 2 [file cddis201720x2.pdf]

Supplementary Figure 2

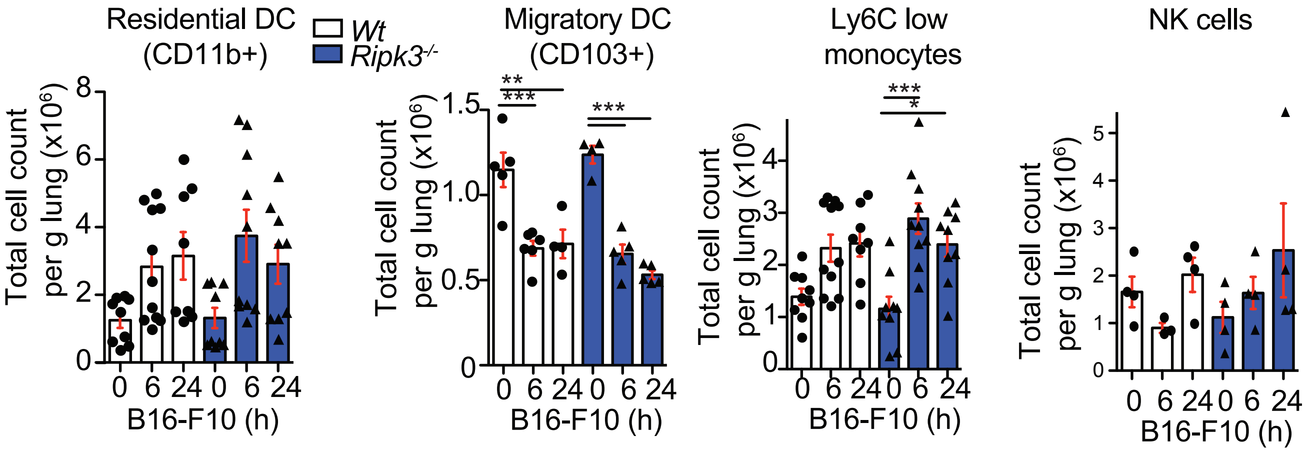

Supplementary Figure 2. Immune cell infiltration analysis of the lung by flow cytometry shows CD11b+ dendritic cells (DC; CD11c+, MHCII+, CD11b+ CD103-), CD103 DC (CD11c+, MHCII+, CD11b- CD103+) and L6C- Monocytes/Macrophages (CD11c-, CD11b+, Ly6C-) and natural killer (NK; NK1.1+ NKp46+) cells at indicated timepoints after B16F10 injection. Populations were pre-gated on singlets, live cells, Ter119- , CD45+ , SiglecF-, Ly6G-). Data pooled of n=2 experiments except NK cells. Each dot represents a mouse; SEM displayed and statistical analysis done by one-way ANOVA and Bonferroni post-test.
