# Supplementary material for: RIPK1/RIPK3 promotes vascular permeability to allow tumor cell extravasation independent of its necroptotic function
Source: Cell Death Dis. 2017 Feb 2;8(2):e2588–. doi: 10.1038/cddis.2017.20 (PMC5386469; doi:10.1038/cddis.2017.20)
Supplement: Supplementary Figure 3 [file cddis201720x3.pdf]

**Supplementary Figure 3**

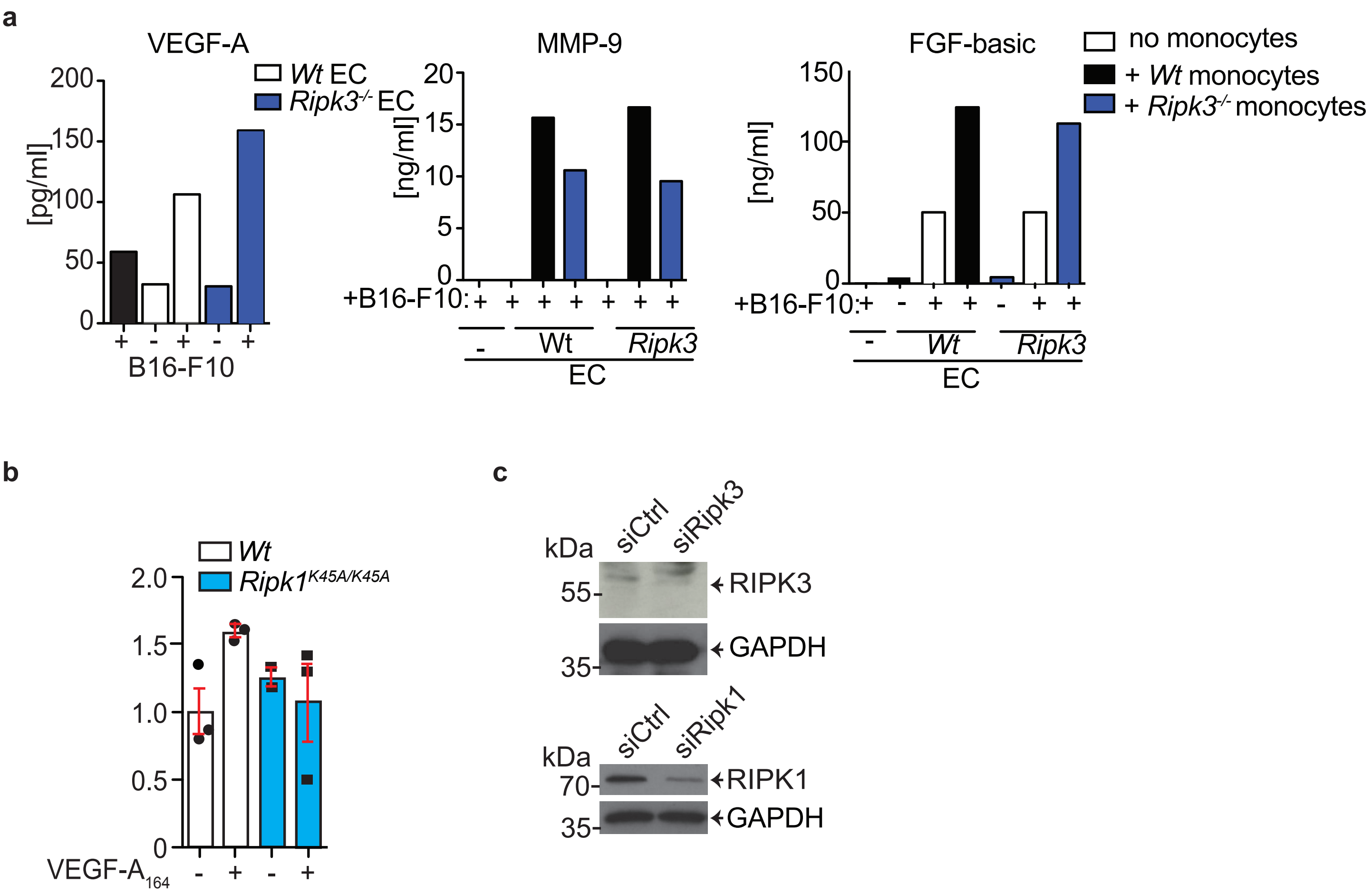


**Supplementary Figure 3. (a)** Protein levels of VEGF-A, FGF basic and MMP-9 were measured from supernatants of transwell migration assays 20h after addition of tumor cells and or monocytes as indicated. **(b)** Primary lung endothelial monolayer on transwell inserts were treated with VEGF-$A_{164}$ (100ng/ml) for 4 hours and dextran-FITC permeability assay was performed. Data shows relative fluorescence unit (RFU) of dextran-FITC that passed EC barrier (SEM; 2-3 transwell inserts were used per group; Each dot represents a transwell insert). **(c)** Immunoblot analysis of RIPK3 and RIPK1 from HUVECs transfected with siRNA targeting *Ripk3* (upper panel) or *Ripk1* (lower panel) from sprouting assays.
