# Supplementary material for: RIPK1/RIPK3 promotes vascular permeability to allow tumor cell extravasation independent of its necroptotic function
Source: Cell Death Dis. 2017 Feb 2;8(2):e2588–. doi: 10.1038/cddis.2017.20 (PMC5386469; doi:10.1038/cddis.2017.20)
Supplement: Supplementary Figure 4 [file cddis201720x4.pdf]

Supplementary Figure 4

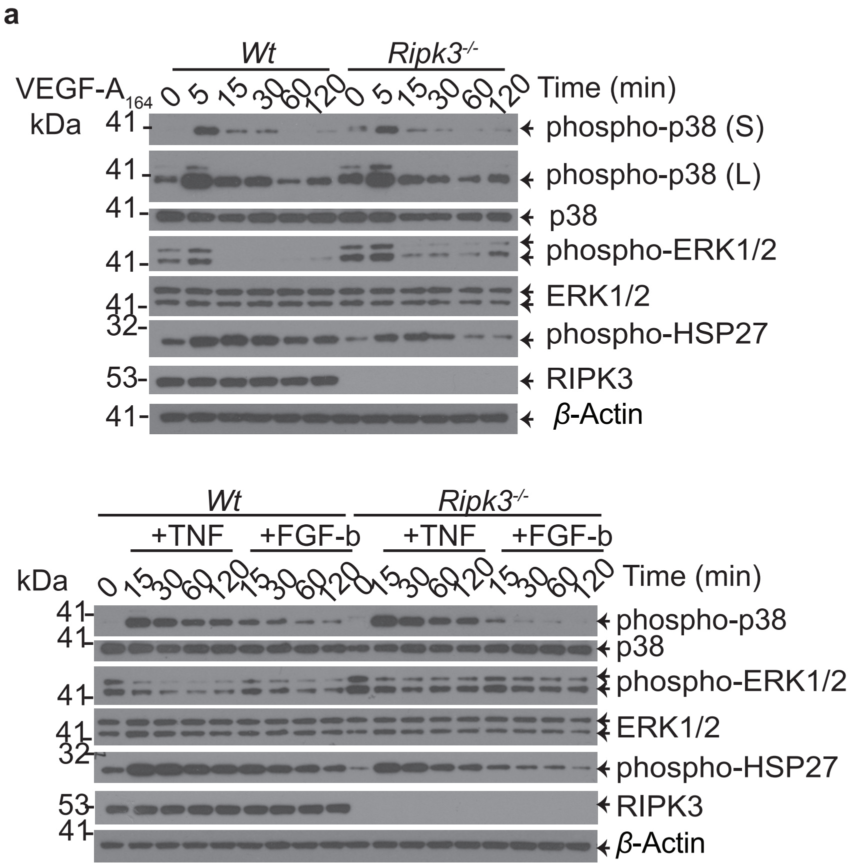

Supplementary Figure 4. **(a)** SV40 large T immortalized endothelial cells isolated from *Wt* or *Ripk3*$^{-/-}$ mice were treated with VEGF-A (10ng/ml), TNF (10ng/ml) or FGF-b (10ng/ml) and assayed for signaling by immunoblot analysis as indicated. Representative immunoblot is shown of n=3 experiments. Statistical analysis by one-way ANOVA and Bonferroni post-test; S=short exposure, L=long exposure. **(b)** Immunoblot quantification of pixel density normalized to total p38 from VEGF-A_{164} or FGF-b treated endothelial cells. Values shown as relative change to untreated *Wt* control. SEM shown and statistical analysis by one-way ANOVA and Bonferroni post-test.
